# Impact of the SARS-CoV-2 Coronavirus Pandemic on Physical Activity, Mental Health and Quality of Life in Professional Athletes—A Systematic Review

**DOI:** 10.3390/ijerph18179423

**Published:** 2021-09-06

**Authors:** Alicja Jurecka, Paulina Skucińska, Artur Gądek

**Affiliations:** 1 Department of Orthopedics and Physiotherapy, Faculty of Health Sciences, Jagiellonian University Medical College, 30-688 Krakow, Poland; artur.gadek@uj.edu.pl; 2 Students’ Scientific Society, Faculty of Health Sciences, Jagiellonian University Medical College, 31-007 Krakow, Poland; paulina_s22@interia.pl

**Keywords:** sportsmen, exercise, training, mental condition, health-related quality of life, COVID-19

## Abstract

Due to the rapid rate of spread of the SARS-CoV-2 coronavirus, a number of restrictions have been introduced into public spaces, including those related to the operation of sports facilities, compounding the difficulty for athletes to conduct appropriate forms of training. The aim of this study was to review current scientific reports assessing the impact of the pandemic on the physical activity, mental state, and quality of life of professional athletes. Popular scientific databases—PubMed, Scopus, and Embase—were systematically searched from the beginning of the pandemic until 12 July 2021. According to the adopted criteria, 14 articles were included in the review. Ten of the qualified studies determined the impact of the pandemic on the physical activity of athletes. The authors of 11 papers assessed the mental state and quality of life of athletes during the pandemic. The studies showed negative effects of the pandemic: a decrease in overall physical fitness and number of days and hours of training, as well as an increase in the occurrence of negative emotions (stress, fatigue, and depression) and a decrease in sleep quality. Changes in physical activity had an impact on overall well-being ratings, which depended on the sex of the subjects. Women were more likely to experience negative emotions compared to men. The mental state of the athletes affected the quality of sleep. This review summarises the negative effects of the SARS-CoV-2 coronavirus pandemic on the physical and mental health of professional athletes.

## 1. Introduction

COVID-19, a disease caused by the SARS-CoV-2 coronavirus, is a serious danger to the health of people all around the world [1]. On the 11th of March 2020, due to a high rate and number of progressing infections with SARS-CoV-2 coronavirus, the World Health Organization (WHO) announced the status of a global pandemic [2]. WHO published the strategy of management-related rules and guidelines in association with the state of the danger of the epidemic in the world. These actions encompassed numerous limitations and restrictions, e.g., among other things, an obligation to keep a social distance and a reduction in the freedom of use of public spaces. The above procedures contributed to withholding both economic and social life [2,3].

Limitations in the sphere of social life, linked to quarantine due to the SARS-CoV-2 coronavirus pandemic, may lead to undesired consequences of a physical and mental nature [4]. Negative results of the isolation cover the following: depression, fear, confusion, and anxiety [5], and also less frequent or even totally deficient episodes of undertaking physical activities [4]. Studies suggest the negative effect of selected mental illnesses and posttraumatic disorders on the immune system functions [6]. In contrast, physical activity belongs to the main neuroprotective and antidepressive factors. During motor exercises, the stimulation of brain-originated factor synthesis takes place, including the brain-derived neurotrophic factor and endorphins, which have a positive effect on the nervous system functions [6]. Health-improving behaviors, including regular physical exercise, play a key part in the normal functions of the circulatory system [6]. Infectious diseases may lead to carditis, increasing the risk of infarction, cardiac insufficiency, and arrhythmia [7]. Regular physical exercise supports circulatory and respiratory efficiency; therefore, it is believed to be necessary during the pandemic [6]. A greater circulatory and respiratory efficiency affects the improvement of pulmonary functions and exerts a directly proportional effect on generating anti-inflammatory reactions [8]. In the case of the movement system, exercises are required for the maintenance of the skeletal muscle’s normal mass [9]. The lack of physical activity leads to anabolic resistance and muscular tissue atrophies [9]. Studies have demonstrated that the worsening of mitochondrial homeostasis at a cellular level produces a local and generalised inflammatory process [6]. The WHO recommends regular physical activity during quarantine and after the cessation of hospital treatment. According to the WHO, physical effort is an inherent element of the convalescence process after combating the COVID-19 infection [10].

In a study [4] from the turn of the second half of 2020, conducted on almost 2.5 thousand Italian citizens, a series of significant differences were observed between the level of weekly physical activity and mental health before and during the pandemic. Reductions in energetic expenses were recorded in all the studied people aged between 21 and 64 years. It was concluded that the drop in motor activity strongly correlates with a worse general feeling. The clinical importance of the above study indicates that the use of regular exercises constitutes a significant prophylaxis for maintaining physical and mental health during the social isolation in connection with the outburst of the SARS-CoV-2 coronavirus pandemic [4]. In turn, Chinese scientists proved that the prevalence of anxiety and depression is almost two-fold greater in people staying in quarantine [11]. The signs above touched predominantly women who had statistically higher scores compared to men. The group of people affected by a compulsory social isolation was characterized by a worse general health condition and an increased fear for the infection with the SARS-CoV-2 coronavirus [11].

A systematic review [12] of May 2020 enabled the formulation of collective reports regarding the effect of the pandemic on the condition of mental health in respective groups of the studied people. Results were processed based on the respondents’ replies, among whom the following people were present: the infected and hospitalised due to the infection with the virus, people with mental disorders, and medical staff. The first study revealed that 96.2% of 714 patients suffered from posttraumatic stress disorder (PTSD). The second case showed that the incidence of depressive symptoms was higher in patients recovered from COVID-19 but subjected to an earlier hospitalization compared to people staying in quarantine [12].

In the light of current scientific reports on the positive impact of physical activity on health [6,8,13,14,15], as well as the negative impact of social isolation during the Covid-19 pandemic on the mental state of different groups of subjects [4,5,11,12], it seems important to include the group of professional athletes in the analysis. Actions taken in many countries to limit the spread of coronavirus included, among others, the suspension of sports facilities [16,17,18]. The inability to undertake regular training with a team of coaches and medics may have significantly affected both the quality of exercise and the psychological state of the athletes. The scientific literature lacks a review paper on the impact of the SARS-CoV-2 coronavirus pandemic on the physical activity or mental health of professional athletes. With regard to the health effects of the pandemic, a given review could be an important position not only for researchers but also for practitioners, including coaches, sports psychologists, physiotherapists, and doctors. This paper is aimed at reviewing the current scientific reports evaluating the effect of the SARS-CoV-2 coronavirus pandemic on physical activity, mental condition, and the quality of life of professional athletes.

## 2. Materials and Methods

This work constitutes a systematic review, and its structure corresponds to the Preferred Reporting Items for Systematic Reviews and Meta-Analyses (PRISMA) guidelines and statement [19].

### 2.1. Search Strategies and Data Source

Detailed systematic research of popular scientific bases, e.g., PubMed, Scopus, and Embase, was carried out from the start of the SARS-CoV-2 coronavirus pandemic until the 12th of July 2021. At the beginning, the authors prepared a synonym list *(elite athletes, players, sportsmen, physical activity, physical fitness, exercise, training, mental state, mental health condition, psychological status, mood state, quality of life, life satisfaction, coronavirus disease, pandemic, epidemic, social isolation, quarantine, sars-cov-2, covid-19)* to define and select appropriate keywords. Any disagreements between review authors were resolved through discussion. The authors of this paper jointly developed the search strategy, which was the same for each database. Details of the search process are presented in Table 1 and Table 2.

Terms such as “epidemic” and “pandemic” were used in line with the definitions suggested by the WHO. According to the WHO’s nomenclature, an epidemic is the occurrence of a disease, defined health-related behavior, or other events in a health-related context that significantly exceed an expected and previous norm in a given population or region [20]. Pandemic is a term defining the spread of a new disease in a global context due to high infectiousness and the lack of natural collective immunity in the population [21].

### 2.2. Eligibility Criteria

Inclusion criteria involved the following: the original research (only full-text articles not being: protocols, reports, and conference abstracts) published in a peer-reviewed journal, age of studied people (>18 years), the professional nature of the practised sport (athletes of national and international leagues only), and the subject coincidence of the review with the study results.

Meta-analyses and review articles were not eligible for the review. The review excluded studies conducted on groups of student athletes who represented university organizations.

The process of eligibility or withdrawal of the studies did not include language, publication date, or methodological quality (e.g., research tools used, statistical methods applied, or form of presentation of results).

### 2.3. Data Extraction

At each stage of the search, all studies were carefully reviewed by the review authors working independently. Data extraction was performed using a Microsoft Excel spreadsheet in accordance with the review eligibility criteria. The form contained the following information: names of the authors of the study, title and type of study, details of the study population, study design, research tools used, and main findings.

### 2.4. Quality Appraisal

The methodological quality and risk of bias of studies eligible for review were assessed using the Joanna Briggs Institute (JBI) critical appraisal tools for cross-sectional [22] and cohort studies [23]. The tool assesses trustworthiness, relevance, and results of published papers [22,23]. The assessment was conducted and verified independently by all review authors. Any disagreements between review authors regarding the qualification and analysis of articles were resolved through discussion. Due to the methodological heterogeneity of studies selected for the review, no statistical calculations defining relationships among the obtained results have been made.

## 3. Results

### 3.1. Search Results

**Figure 1:**
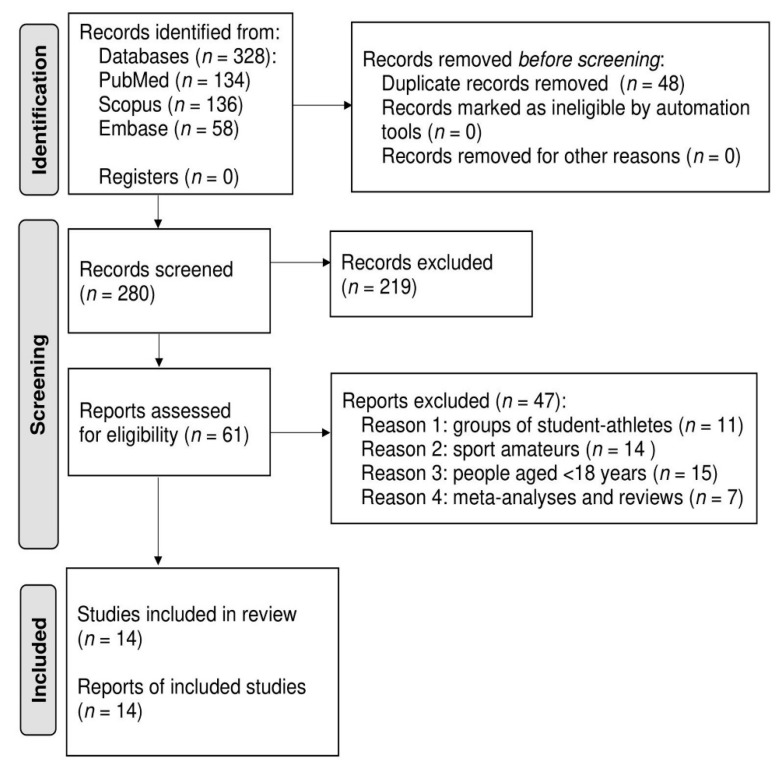
PRISMA flow diagram to depict search strategy results.

The search of all three scientific bases resulted in the generation of (328) articles. After the removal of duplicated papers (48), studies were left for verification. The final inclusion criterion for the review was met by 14 articles [16,24,25,26,27,28,29,30,31,32,33,34,35,36]. Details were presented in the PRISMA diagram [19] (Figure 1).

The articles qualified for the review included a total of 5434 respondents. The female group (n = 2742) represented the majority of subjects (51%), while the male group (n = 2692) represented a total of 49%. The athletes’ age was included in 13 articles [16,24,25,26,27,28,30,31,32,33,34,35,36]. Only seven studies [16,25,26,27,28,30,32] included a comparison of the results obtained in times before and during the pandemic, while only one research article [30] characterized the results in relation to the different phases of the pandemic. Of all the articles, only three referred to the subjects’ history of SARS-CoV-2 coronavirus infection [27,30,31].

### 3.2. Quality Appraisal Findings

Among 8 cross-sectional studies included in the review, 2 articles (25%) [24,34] were of very good quality, 3 articles (38%) [30,31,36] were of good quality, and 3 articles (38%) [29,33,35] were of average quality. Of the 6 cohort studies, 4 articles (67%) [16,27,28,32] were of good quality and 2 articles (33%) [25,26] were of average quality. Details of the study characteristics and evaluation are presented in Table 3, Table 4, and Table 5.

### 3.3. Risk of Bias Assessment

Only published articles were included in the review, and grey literature was excluded, which could potentially increase the risk of publication bias. Additionally, the use of an electronic survey questionnaire as a research tool may have influenced the response bias and systematic bias.

**Table 3:** Study characteristics.

Author, Year	Sample Characteristics				P-A/M-H Outcome Measures	Main Findings (pre/during the Pandemic)
	Sports Discipline/League	n	n f:m	Age (Years)		
Hermassi S., 2021 [16]	Handball	1359	463:896	23 ± 6	The International Physical Activity Questionnaire (IPAQ-SF)	During the pandemic: ↓ in P-A (d = 2.89; *p* < 0.05); pre vs. during the pandemic: 4303 ± 908 vs. 1264 ± 647 METs per week; in f: ↓ by 2863 METs, and in m: ↓ by 3129 METs (*p* < 0.001); during the pandemic: ↑ in time spent in a sedentary position by a mean of 2.3 h per average day (d = 1.89; *p* < 0.001)
Donmez G., 2021 [24]	Football	237	0:237	27.2 ± 5.3	The International Physical Activity Questionnaire (IPAQ);	During the pandemic: high levels of P-A, 3756.4 ± 2330.7 METs per week; the vast majority of football players (82.3%) had a high level of physical activity;
					The Center of Epidemiologic Studies Depression Scale (CES-D);	During the pandemic: 9.1 ± 9.6 points (highest score obtained: 42 points), which indicated the presence of depressive symptoms;
					The Impact of Event Scale-Revised (IES-R)	During the pandemic: 24.3 ± 11.5 points (highest score: 59 points), which indicated the presence of post-traumatic stress disorder (PTSD) symptoms
Villaseca-Vicuna R., 2020 [25]	Soccer	32	32:0	26 ± 4	The Subjective Effort Perception (SEP);	During pandemic: no significant changes in training load (%) and duration (min) of a given activity under pandemic conditions;
					McLean Screening Instrument for Borderline Personality Disorder (MSI-BPD)	During the pandemic: ↓ in M-H condition (d = 2.4; *p* < 0.05); pre vs. during the pandemic: 19.3 ± 0.6 vs. 18.2 ± 0.2 points
Urbański P., 2021 [26]	Athletics (23.5%)	166	66:106	33 ± 11.7	Amount of training hours per week	During the pandemic: ↓ in training hours per week (*p* < 0.001); pre vs. during the pandemic: 9.4 vs. 5.3 h per week; during the pandemic: 12% of respondents completely suspended exercise, over 74% of respondents had a low level of satisfaction with their training opportunities, and only 5.4% of respondents declared the use of sports facilities
Mon-Lopez D., 2020 [27]	Football	175	25:150	f: 24.32 ± 4.55 m: 25.89 ± 5.23	Amount of training hours and days per week;	During the pandemic: ↓ in training hours (d = −0.82; *p* < 0.001) and days (d = −0.27; *p* < 0.05) per week; pre vs. during the pandemic: 10.82 ± 4.87 vs. 7.26 ± 4.38 h and 5.18 ± 1.05 vs. 4.91 ± 1.43 days per week, in f: 12 ± 4.87 vs. 8.64 ± 4.21 h, in m: 10.63 ± 4.85 vs. 7.03 ± 4.38 h, and 5.16 ± 1 vs. 4.91 ± 1.44 days per week (*p* < 0.05);
					The Profile of Mood States (POMS); The Wong Law Emotional Intelligence Scale (WLEIS-SF)	During the pandemic: ↓ sleep quality (d = −0.51; *p* < 0.001), m had higher sleep quality scores than f (*p* < 0.05), high scores of: depression, anger, fatigue, and tension, POMS scores had a significant (*p* < 0.05) effect on sleep quality in m (*r* = 0.18–0.37), and on the number of hours of sleep in f (*r* = 0.47–0.55)
Facer-Childs E.R., 2021 [28]	Football, field hockey, and netball	440	252:188	26.4 ± 8.7	Amount of training hours and days per week;	During the pandemic: ↓ in training hours and days per week (*p* < 0.001); pre vs. during the pandemic: 1–2 vs. <1 h (single training session), and 5.3 vs. 4.3 training days per week;
					The Patient Health Questionnaire-4 (PHQ-4); The Perceived Stress Scale-4 (PSS-4);	During the pandemic: ↑ in negative emotions: depression, anxiety, and stress (*p* < 0.05);
					The Morningness-Eveningness Questionnaire-19 (MEQ-19)	During the pandemic: ↑ in time spent in bed by 0.8 h, ↑ in sleep time by 0.6 h, ↑ in sleepiness during an average day (*p* < 0.05), ↑ in mean delay in falling asleep in subjects who spent less time in front of electronic devices by 22 min and in subjects who spent more time in front of electronic devices by 37 min (*p* < 0.05)
Pillay L., 2020 [29]	Football, hockey, and rugby	692	229:463	N/S	The Physical Activity Readiness Questionnaire (PAR-Q);	During the pandemic: 61% of subjects trained daily only at moderate intensity for 30–60 min, P-A was more frequently undertaken by m than f (*p* < 0.05), in free time: ↑ in sedentary activities (*p* < 0.05);
					The Diagnostic and Statistical Manual of Mental Disorders (DSM-5)	During the pandemic: 52% of all athletes experienced negative emotions, and higher rates of depression, lack of energy, and motivation were reported among female subjects (*p* < 0.05)
Mehrsafar A.H, 2021 [30]	Super league, national, and international levels	I 525; II 464; III 428	I 320:205; II 281:183; III 259:169	I 27.85 ± 9.09; II 27.53 ± 8.74; III 27.43 ± 8.56	Amount of training hours per week;	Pre-pandemic: 11.02 ± 8.58 h per week; During three phases of pandemic: I, II, III: ↓ in training hours per week (*p* < 0.001); I vs. II vs. III: 1.34 ± 1.14 vs. 1.65 ± 1.2 vs. 1.51 ± 1.07 h per week;
					The General Health Questionnaire-28 (GHQ-28); The Satisfaction with Life Scale (SWLS); The Profile of Mood States (POMS)	During three phases of pandemic: I—↓ in M-H condition and life satisfaction (*p* = 0.001) and II, III—↑ in M-H condition and life satisfaction (*p* = 0.001); GHQ-28 I vs. II vs. III: 45.1 ± 5.89 vs. 35.38 ± 5.13 vs. 41.82 ± 5.25 points; SWLS I vs. II vs. III: 16.18 ± 4.61 vs. 17.34 ± 4.18 vs. 18.65 ± 5.48 points; depression I vs. II vs. III: 8.66 ± 0.47 vs. 7.41 ± 0.49 vs. 7.59 ± 0.49 points, and fatigue I vs. II vs. III: 8.33 ± 0.85 vs. 7.42 ± 0.63 vs. 8.08 ± 0.76
Parm U., 2021 [31]	Athletics (22.6%)	102	58:44	24.68 ± 8.55	Training conditions;	During the pandemic: 34.3% of the subjects continued their training program at the pre-pandemic level; 43.1% of subjects attempted to maintain their fitness level; 4.9% of the athletes ceased physical activity altogether;
					The Emotional State Questionnaire (EST-Q2)	During the pandemic: ↑ in negative emotions: fatigue, depression, insomnia, and anxiety; f were found to have more symptoms of fatigue and insomnia (*p* < 0.05)
Ambroży T., 2021 [32]	Kickboxing	20	0:20	25.2 ± 3.02	Respiratory capacity (VO_2_ max), time during a 50 m sprint, distance jump length from a standing position, time during a 1000 m run, grip strength (measured with a dynamometer (under straight arm conditions), number of pull-ups per time unit during a pull-up, time during a 5 × 10 m shuttle run, number of squats in 30 s, range of motion during a forward bend, and the BMI (kg/m^2^)	During the pandemic: ↓ in mean values of all tests; statistically significant differences (*p* < 0.05) in each of the tests performed except: 50 m sprint, grip strength, and squats; the largest measurement effect size was observed for the forward bend (d = 1.36) and pull-up bar (d = 0.61); ↑ in athletes’ BMI (d = 0.75; *p* < 0.001); athletes’ BMI pre vs. during the pandemic: 25.27 ± 1.09 vs. 26.08 ± 1.13 kg/m^2^; the change in BMI only affected the distance jump score (r = −0.45; *p* < 0.05)
Roberts R.J., 2021 [33]	Boxing	44	11:33	19.4 ± 4.6	The Brunel Mood Scale (BRUMS)	During the pandemic: ↑ in negative emotions: anger, confusion, fatigue, tension, depression, and ↓ in vigor (d = 0.93; *p* < 0.01); pre vs. during the pandemic: anger 2.02 ± 2.16 vs. 5.29 ± 3.84, confusion 1.43 ± 2.31 vs. 6.61 ± 4.01, depression 1.04±1.81 vs. 4.47 ± 3.90, fatigue 2.52 ± 2.37 vs. 3.36 ± 3.24, tension 1.27 ± 1.78 vs. 3.20 ± 3.12, vigor 10.64 ± 3.44 vs. 7.81 ± 3.91
Jaenes Sanchez J.C., 2021 [34]	Swimming	1248	661:587	22.31 ± 11.49	The Recovery–Stress Questionnaire for Athletes (RESTQ-Sport); The Profile of Mood States (POMS)	During the pandemic: ↓ in M-H condition; women reported higher stress symptoms (*p* < 0.05), female subjects had higher ratings of negative emotions (*p* < 0.001), depression f vs. m: 2.08 ± 0.93 vs. 1.87 ± 0.87 points, and anger f vs. m: 2.13 ± 0.81 vs. 1.91 ± 0.75 points
di Cagno A., 2020 [35]	Athletics, basketball, volleyball, and football	671	297:374	27.59 ± 10.73	The Impact of Events Scale-Revised (IES-R)	During the pandemic: 29.81% of participants had total IES-R scores higher than the accepted norm (43.37 ± 9.84 points), female subjects scored higher on the total score (44.54 ± 9.74 points) and avoidance subscale (15.21 ± 3.84 points), higher scores on the hyperarousal subscale were reported among top league athletes, who had 11.62 ± 3.89 points (*p* < 0.05), significant differences were observed among individually trained athletes, who had a total score of 45.2 ± 11.09 points (*p* < 0.05)
Fiorilli G., 2021 [36]	Local and international competitors	800	374:426	28.25 ± 10.97	The Impact of Event Scale-Revised (IES-R)	During the pandemic: (31.2%) athletes were affected by symptoms of severe stress (≥33 points), women were more likely to report stress symptoms (*p* < 0.05), lower scores of the total IES-R score were obtained by team athletes (d = 0.213; *p* < 0.05), mid-league athletes were found to have higher scores in the hyperarousal subscale (d = 0.223; *p* < 0.05)

BMI—body mass index, d—effect size, f—females, m—males, M-H—Mental Health, n—number of participants, N/S—not stated, *p*—value, P-A—Physical Activity, r—correlation coefficient, ↓—decrease, ↑—increase, I—total restrictions due to the pandemic, II—reopening condition, III—semi-lockdown phase.

**Table 4:** Critical appraisal of cross-sectional studies.

Author, Year	Were the Criteria for Inclusion in the Sample Clearly Defined?	Were the Study Subjects and the Setting Described in Detail?	Was the Exposure Measured in a Valid and Reliable Way?	Were Objective, Standard Criteria Used for Measurement of the Condition?	Were Confounding Factors Identified?	Were Strategies to Deal with Confounding Factors Stated?	Were the Outcomes Measured in a Valid and Reliable Way?	Was Appropriate Statistical Analysis Used?	Johanna Briggs Institute Score
Donmez G., 2021 [24]	✓	✓	✓	✓	✓	✓	✓	✓	8
Pillay L., 2020 [29]	✓	X	✓	✓	✓	X	✓	✓	6
Mehrsafar A.H, 2021 [30]	✓	✓	✓	✓	✓	X	✓	✓	7
Parm U., 2021 [31]	✓	✓	✓	✓	✓	X	✓	✓	7
Roberts R.J., 2021 [33]	X	✓	✓	✓	✓	X	✓	✓	6
Jaenes Sanchez J.C., 2021 [34]	✓	✓	✓	✓	✓	✓	✓	✓	8
di Cagno A., 2020 [35]	✓	✓	✓	✓	✓	X	✓	X	6
Fiorilli G., 2021 [36]	✓	✓	✓	✓	✓	X	✓	✓	7

✓—Yes, X—No.

**Table 5:** Critical appraisal of cohort studies.

Author, Year	Were the Two Groups Similar and Recruited from the Same Population?	Were the Exposures Measured Similarly to Assign People to Both Exposed and Unexposed Groups?	Was the Exposure Measured in a Valid and Reliable Way?	Were Confounding Factors Identified?	Were Strategies to Deal with Confounding Factors Stated?	Were the Groups/Participants Free of the Outcome at the Start of the Study (or at the Moment of Exposure)?	Were the Outcomes Measured in a Valid and Reliable Way?	Was the Follow-Up Time Reported and Sufficient to Be Long Enough for Outcomes to Occur?	Was Follow-Up Complete, and If Not, Were the Reasons for Loss to Follow-Up Described and Explored?	Were Strategies to Address Incomplete Follow Up Utilized?	Was Appropriate Statistical Analysis Used?	Johanna Briggs Institute Score
Hermassi S., 2021 [16]	X	N/A	✓	✓	✓	✓	✓	✓	✓	N/A	✓	8
Villaseca-Vicuna R., 2020 [25]	X	N/A	✓	X	N/A	✓	✓	✓	✓	N/A	✓	6
Urbański P., 2021 [26]	X	N/A	✓	✓	X	✓	✓	✓	✓	N/A	✓	7
Mon-Lopez D., 2020 [27]	X	N/A	✓	✓	✓	✓	✓	✓	✓	✓	✓	9
Facer-Childs E.R., 2021 [28]	X	N/A	✓	✓	✓	✓	✓	✓	✓	✓	✓	9
Ambroży T., 2021 [32]	X	N/A	✓	✓	✓	✓	✓	✓	✓	N/A	✓	8

✓—Yes, X—No, N/A—Not applicable.

### 3.4. Assessment of Physical Activity during the SARS-CoV-2 Coronavirus Pandemic

Ten articles were identified reporting the impact of the COVID-19 pandemic on athletes’ physical activity [16,24,25,26,27,28,29,30,31,32]. The authors of four articles did not use standardised scales or questionnaires to assess physical activity [26,27,28,30]. Estimates of physical activity levels during the pandemic were based on the number of training days and hours [26,27,28,30] and using comprehensive cardiac stress tests [32]. The remaining studies used appropriate survey instruments: the original and abbreviated versions of the International Physical Activity Questionnaire (IPAQ and IPAQ-SF) [16,24], the Subjective Effort Perception (SEP) [25] measure, and the Physical Activity Readiness Questionnaire (PAR-Q) [29] (Table 3).

#### 3.4.1. Overall Physical Activity

Hermassi S. et al. [16] and Donmez G. et al. [24] in their study evaluated the level of overall physical activity of athletes in the pandemic. In a study [16] conducted on a group of handball players from Europe, North Africa, and Western Asia, there was a decrease in the overall physical activity of athletes during the pandemic. On the other hand, the studies of Donmez G. et al. [24] demonstrate that the vast majority of football players (82.3%) had a high level of physical activity. However, the results of the study were not compared with the pre-pandemic period or between specific pandemic periods.

#### 3.4.2. Training Days and Hours

The most common measure of physical activity in the study was the number of exercise days and exercise hours per week [26,27,28,29,30]. The authors of most studies [26,27,28,30] demonstrated that there was a reduction in training days and hours during the pandemic. Pillay L. et al. [29] observed that the majority of subjects (61%) trained only at moderate intensity for 30–60 min daily, and physical activity was more frequently undertaken by men than women. Additionally, in the Mon-Lopez study [27] differences in the values of the analyzed indicators were observed according to sex. In women, the pandemic significantly affected only the number of training hours, while in men, the pandemic period resulted in a reduction in both the number of days and hours of training [27]. Additionally, Facer-Childs et al. [28] demonstrated that a reduction in training frequency during the pandemic was strongly associated with increased depression, anxiety, and stress scores. Mehrsafar A. et al. [30] assessed the training rate (number of hours per week) during three phases of the pandemic: total restrictions due to the pandemic (14–24 April 2020), the reopening condition (9–19 May 2020), and the semi-lockdown phase (20–31 July 2020). It was determined that the stated rate was higher during the reopening condition compared to the total restrictions and the semi-lockdown phase during the pandemic [30].

#### 3.4.3. Physical Fitness

Ambroży T. et al. [32] determined the effect of the COVID-19 pandemic on the training activity in kickboxers using comprehensive cardiac stress tests. Additionally, the BMI (kg/m^2^) of the subjects was determined. It was noted that all test scores deteriorated in the pandemic, but the greatest differences were observed in the measurements of standing long jump, 1000 m run, pull up, 4 × 10 m shuttle run, and forward bend. It was observed that the change (increase) in BMI during the pandemic only affected the distance jump score [32]. In turn, Villaseca-Vicuna R. et al. [25] evaluated the training load in players of the Chilean football team. The result indicated that there were no significant changes in training load (%) or duration (min) of a given activity under pandemic conditions [25].

#### 3.4.4. Training Conditions

According to a study by Parm U. et al. [31], the most distressing issues for athletes were the closure of training centers (57.8%) and the cancellation of competitions (50%)—associated with the European Championships (36.3%) or the Olympic Games (19.6%). Loss of previous training opportunities (43.1%) and unknown professional future (17.6%) also worried the athletes. The majority of participants (52%) found the alternative training conditions interesting/challenging, while 21.6% of the athletes rated the new conditions as tiring. It was demonstrated that during the pandemic, 34.3% of the subjects continued their training program at the pre-restriction level, 43.1% attempted to maintain their fitness level, and 4.9% of the athletes ceased physical activity altogether. The lack of proper training conditions and absence of sports coaches had a negative impact on the fluidity and regularity of training for 9.8% of the respondents; 32.4% of the respondents declared that the absence of a coach had no impact on their training process, and for the others, the impact was moderate [31].

#### 3.4.5. Sedentary Behavior

Two researchers [16,29] evaluated changes in sedentary lifestyle during the COVID-19 pandemic. In a study by Hermassi S. et al. [16], it was shown that the length of time spent sitting during an average day at the time of the pandemic increased for the whole sample. Furthermore, Pillay L. et al. [29] observed that in their free time during the pandemic, subjects were more likely to choose sedentary activities, e.g., watching TV or computer games, over active recreation.

### 3.5. Mental State Assessment and Quality of Life of Athletes during the SARS-CoV-2 Coronavirus Pandemic

Mental state and quality of life with used standardized scales and questionnaires were assessed in 11 studies [24,25,27,28,29,30,31,33,34,35,36]. The following research tools were applied: the Centre of Epidemiologic Studies Depression Scale (CES-D) [24], the Impact of Events Scale-Revised (IES-R) [24,35,36], the McLean Screening Instrument for Borderline Personality Disorder (MSI-BPD) [25,37], the Profile of Mood States (POMS) [27,30,34], a shortened version of the Wong Law Emotional Intelligence Scale (WLEIS-SF) [27], the Patient Health Questionnaire-4 (PHQ-4) [28], the Perceived Stress Scale-4 (PSS-4) [28], the Morningness–Eveningness Questionnaire-19 (MEQ19) [28], the Diagnostic and Statistical Manual of Mental Disorders (DSM-5) [29], the General Health Questionnaire (GHQ-28) [30], the Satisfaction with Life Scale (SWLS) [30], the Emotional State Questionnaire (EST-Q2) [31], the Brunel Mood Scale (BRUMS) [33], and the Recovery–Stress Questionnaire for Athletes (RESQ-Sport) [34] (Table 3).

#### 3.5.1. General Mental State Condition

Three researchers [25,30,34] determined the impact of the COVID-19 pandemic on the overall mental state of professional athletes. The results of all the studies proved that during the pandemic, the point value of the athletes’ total mental state assessment decreased. Furthermore, in a study by Mehrsafar A. et al. [30], it was concluded that the reopening condition and the semi-lockdown phase were associated with better emotional health [30]. Studies performed by Jaenes-Sanchez J.C. et al. [34] presented the assessment of mental status depending on the sex of the subjects. It was noted that female subjects had higher ratings of negative emotions compared to male subjects. There were no significant differences in the assessment of positive emotions between women and men. For both men and women, training conditions during the pandemic were positively associated with negative emotions and negatively associated with positive emotions [34].

#### 3.5.2. Depression, Tension, and Anxiety

The most frequently rated emotions included depression, tension, and anxiety [24,27,28,29,31,33]. All studies confirmed a significant increase in negative emotions during the pandemic. Moreover, a study by Pillay L. et al. [29] reported that as many as 52% of all athletes experienced negative emotions, while significantly higher rates of depression were reported among female subjects. In turn, Parm U. et al. [31] noted that those who described the pandemic conditions as an opportunity to train and prepare in unique ways were significantly less likely to have depressive symptoms.

#### 3.5.3. Stress

The stress levels of professional athletes have been evaluated in several articles [34,35,36]. Studies have confirmed a significant increase in stress indicators during the COVID-19 pandemic. Study authors [34,35,36] noted a significant relationship between stress indicators and the sex of the subjects. It was noted that women reported higher stress symptoms compared to men [34,35,36]. Additionally, women had higher scores on intrusion (INT), avoidance (AV), and hyperarousal (HYP) subscales compared to men [36]. Furthermore, it was shown that lower scores of the total IES-R score were obtained by team athletes compared to individually trained athletes [35,36]. Additionally, significantly higher scores on the HYP subscale were reported among top league athletes [35]. In turn, in studies performed by Fiorilli G. et al. [36], mid-league athletes were found to have higher scores in the HYP subscale compared to upper-league athletes. Studies performed by Jaenes-Sanchez J.C. et al. [34] reported that appropriate training conditions and trainer support were found to be associated with reduced levels of anxiety and stress.

#### 3.5.4. Fatigue and Insomnia

The authors of a number of papers have assessed the fatigue and insomnia levels of athletes during the pandemic [27,28,29,31,33]. All studies reported increased rates of fatigue and insomnia during the pandemic. Studies [27,29,31] reported differences in results depending on sex. In studies performed by Pillay L. et al. [29], it was noted that female athletes more often reported a lack of energy and motivation compared to male subjects. Additionally, women were found to have more symptoms of fatigue and insomnia compared to men [29,31]. Mon-Lopez D. et al. [27] demonstrated that the pandemic period reduced sleep quality among the subjects, while men had higher sleep quality scores than women. It was reported that the POMS scores had a significant effect on sleep quality in men, while in women, it had a significant effect on the number of hours of sleep [27]. Additionally, a study by Parm U. et al. [31] noted that athletes who found the pandemic situation exhausting had higher rates of insomnia. In turn, studies performed by Facer-Childs E.R. et al. [28] demonstrated that the length of time spent in bed and the length of sleep time increased during the pandemic. Despite the increase in sleep time, athletes reported increased sleepiness during an average day. Spending time in front of an electronic device before going to bed caused a delay in falling asleep among respondents. Later bedtimes during the COVID-19 pandemic were significantly associated with poorer mental state outcomes for athletes [28].

#### 3.5.5. Quality of Life

Mehrsafar A. et al. [30] demonstrated that quality of life was significantly influenced by the phases of the COVID-19 pandemic. It was concluded that the reopening condition and the semi-lockdown phase were associated with life satisfaction [30].

## 4. Discussion

Competitive sports, encompassing a wide range of professional and amateur disciplines, are one of the professional sectors that were clearly affected by the COVID-19 pandemic [38]. The given review aims to present the effects of the SARS-CoV-2 coronavirus pandemic in terms of a group of professional players. The studies discussed in the review proved that the pandemic had a negative impact on parameters of physical activity and well-being. During the pandemic, there was a reduction in the number of training days and hours [26,27,28,30], which resulted in a deterioration in the overall activity [16] and physical performance [32] of the athletes. Furthermore, time spent in a sedentary position was significantly increased during the pandemic [16,29]. During the pandemic, a significant deterioration in psychological health [24,25,27,28,29,30,31,33,34,35,36] and life satisfaction [30] was also noted. A detailed analysis of the results distinguished variables regarding the athletes’ sex and occupational ranking. Available studies suggest that during the pandemic, men were more likely to engage in physical activity compared to women [16,29]. During the COVID-19 pandemic, upper-division athletes had higher levels of physical activity compared to lower-division athletes [27]. Sources suggest that during the pandemic, a higher proportion of subjects with symptoms of depression, anxiety, and emotional disturbance were women [29,34,35,36]. The study noted that during the pandemic, men had better sleep quality compared to women [27].

### 4.1. The Level of Athletes’ Physical Activity during the SARS-CoV-2 Coronavirus Pandemic

The authors of a number of studies [16,24,25,26,27,28,29,30,31,32] have assessed the physical activity levels of professional athletes during the pandemic. Most of them proved that the SARS-CoV-2 coronavirus pandemic had a significant impact on reducing the total physical activity of athletes [16,26,27,28,29,30,32]. Only Villaseca-Vicuna R. et al. [25] and Donmez G. et al. [24] obtained different findings. They showed that the training load of Chilean soccer players did not change during the pandemic, and athletes regularly undertook physical activity [25]. In addition, Turkish athletes reported high levels of sporting activity during the COVID-19 pandemic [24].

The different results of the studies may be related to differences regarding the restrictions, concerning the sport and recreation sectors during the SARS-CoV-2 coronavirus pandemic in the countries where the studies were conducted. In addition, details of the pandemic period during which the studies were conducted may also have had a significant impact on the outcome of the studies. The possibility of contact with the trainer may also have been crucial. The duration of mandatory social isolation, during which athletes did not have the opportunity to undertake routine training with a coach under convenient conditions, varied in many countries around the world [39]. According to the study of Urbański P. et al. [26], a significant minority of athletes (5.4%) had the opportunity to train in sports facilities. It was further proved that the support of a coach is a significant factor influencing the reduction of negative emotions, including stress or behavioral problems and increased motivation to undertake physical activity [40]. In their study, di Fronso S. et al. [40] noted that 80% of Italian athletes were in contact with a coach during the pandemic. Sources indicate that the vast majority of respondents expressed concern about their own future in sport [41]. In light of the research results obtained by many authors, it seems extremely important to develop new methods of communication between coaches and athletes in order to be able to supervise the level of physical activity and the way athletes conduct their individual training [42,43].

### 4.2. Mental Health and Quality of Life of Athletes during the SARS-CoV-2 Coronavirus Pandemic

Numerous studies [24,25,27,28,29,30,31,33,34,35,36] have assessed the impact of the COVID-19 pandemic on the emotional state and quality of life of elite athletes. The results obtained proved that mental health indicators and life satisfaction deteriorated significantly during the pandemic [24,25,27,28,29,30,31,33,34,35,36]. The results of all authors cited in this regard were consistent with each other.

Social isolation due to the SARS-CoV-2 coronavirus pandemic had significant effects on many levels of athletes’ lives, including physically and mentally. A reduction in the level of physical activity undertaken or a complete cessation of training can reduce the physiological adaptive and neuromuscular capacities of athletes, thereby increasing the risk of injury [44,45]. Being in quarantine as a result of SARS-CoV-2 coronavirus infection has been compared to prolonged athlete detraining [4,46]. This process leads to a reduction in the body’s maximal oxygen consumption (VO_2_ max.), a decrease in overall endurance capacity, a loss of strength and muscle mass, and an increase in the likelihood of musculoskeletal damage [4]. There are strategies that can have a positive impact on an athlete’s effective return to their sport. One of these strategies is specific strength training, which even in pandemic conditions seems feasible [47].

It is important to remember the highly significant impact of quarantine on the mental health of athletes. According to the available literature, individuals in social isolation due to COVID-19 disease had a two-fold higher prevalence of depression and anxiety compared to those who were not in quarantine [11]. For this reason, the diagnosis of disorders and distressing symptoms is particularly important. Appropriate management, e.g., psychotherapy or pharmacological treatment, will help to mitigate the negative effects of the SARS-CoV-2 coronavirus pandemic [48].

### 4.3. Strengths and Limitations of the Review

The strength of this paper is that it is a review of current scientific reports, consisting of publications from the past year. In total, the study consisted of responses from a very large number of athletes (n = 5434) from around the world: Europe, Western Asia, and South Africa. The research tools consisted of anonymous, standardized questionnaires and scales with appropriate validations [16,24,25,27,28,29,30,31,33,34,35,36]. The study had the approval of the Bioethics Committees and was conducted in accordance with the Declaration of Helsinki.

Potential limitations of this review are the still small number of cohort studies and scientific reports on the effects of the SARS-CoV-2 coronavirus pandemic in terms of groups of elite athletes, the methodological variability of the studies, and the recurrent lack of data on physical and mental health parameters of athletes before the pandemic began. In addition, only published articles were included in the review, and grey literature was excluded, which could potentially increase the risk of publication bias. Another limitation may be that the authors of many of the papers [16,24,26,27,28,29,30,31,33,34,35,36] selected for the review used an electronic survey questionnaire as a research tool, which may have influenced the response bias and systematic bias. However, in the current pandemic situation, the use of objective and direct research methods was not advisable. Due to the methodological differences of the studies, the authors of this review did not attempt a meta-analysis.

## 5. Conclusions

This review summarises the negative effects of the SARS-CoV-2 coronavirus pandemic on the physical and mental health of professional athletes. Among the most commonly observed effects are decreased physical activity, increased time spent sedentary, increased negative emotions (stress, fatigue, anger, tension, and depression), and decreased sleep quality. Temporary restriction banning access to sports facilities was associated with a decrease in overall physical fitness and the number of days and hours of training. Changes in training conditions had a detrimental effect on athletes’ mental state. Women were more likely to experience unpleasant emotions compared to men. Negative mood states accompanying the pandemic affected the quality of sleep and significantly delayed falling asleep.

## 6. Practical Application

According to the results of the study, it seems appropriate to develop alternative, virtual forms of communication between coaches and athletes, which could support professional athletes during the COVID-19 pandemic. In order to improve the quality of training, mental health indicators, and life satisfaction of athletes during the pandemic, it is recommended to develop guidelines related to the motivation of athletes and the conduct of training outside sports facilities. The team of specialists working with athletes should pay special attention to injury prevention and the mental state of athletes during the pandemic.

In the future, more large-scale studies should be conducted to confirm the existence of a strong relationship between the sex and sporting rank of the subjects and indicators of physical activity and mental state. These studies would allow us to exclude potential confounding factors binding conclusions on the above relationships.

## Figures and Tables

**Table 1:** Characteristics of qualitative data and search criteria based on the PICO model.

PICOC	Description
Population	Elite and semi-professional athletes (national and international leagues), female and male aged over 18 years
Exposure	The status of a global pandemic and its consequences: social isolation, quarantine, banning access to sports facilities (no organised team training with a coach)
Comparison	Period before or during specific phases of the pandemic (total restrictions, reopening condition, semi-lockdown)
Outcomes	The effects of the pandemic include but are not limited to decreased physical activity and life quality and mental state deterioration (increased rates of depression, anxiety, and fatigue)

**Table 2:** Key words used in the process of searching in the PubMed, Scopus, and Embase databases.

Search Number—Database	Keywords Combination
1—PubMed	“physical activity”, “mental health”, “quality of life”, “athletes”, “coronavirus”, “pandemic”, “epidemic”, “sars-cov-2”, “covid-19”
2—Scopus	“physical activity” AND “mental health” AND “quality of life” AND “athletes” AND “coronavirus” AND “pandemic” OR “epidemic” AND “sars-cov-2” OR “covid-19”
3—Embase	“physical activity” AND “mental health” AND “quality of life” AND “athletes” AND “coronavirus” AND “pandemic” OR “epidemic” AND “sars-cov-2” OR “covid-19”
Sum of databases searches	PubMed AND Scopus AND Embase

## Data Availability

The data presented in this study are available at online version.

## References

[1] Rothan H.A., Byrareddy S.N. (2020). The epidemiology and pathogenesis of coronavirus disease (COVID-19) outbreak. J. Autoimmun..

[2] Włodarczyk W.C. (2020). Remarks on COVID-19 Pandemic in Poland. A Health Policy Perspective. Public Health Manag..

[3] World Health Organization COVID-19 Strategy Update. https://www.who.int/docs/default-source/coronaviruse/covid-strategy-update-14april2020.pdf?sfvrsn=29da3b.

[4] Maugeri G., Castrogiovanni P., Battaglia G., Pippi R., D’Agata V., Palma A., Di Rosa M., Musumeci G. (2020). The impact of physical activity on psychological health during Covid-19 pandemic in Italy. Heliyon.

[5] Brooks S.K., Webster R.K., Smith L.E., Woodland L., Wessely S., Greenberg N., Rubin G.J. (2020). The psychological impact of quarantine and how to reduce it: Rapid review of the evidence. Lancet.

[6] Woods J.A., Hutchinson N.T., Powers S.K., Roberts W.O., Gomez-Cabrera M.C., Radak Z., Berkes I., Boros A., Boldogh I., Leeuwenburgh C. (2020). The COVID-19 pandemic and physical activity. Sports Med. Health Sci..

[7] Inciardi R.M., Lupi L., Zaccone G., Italia L., Raffo M., Tomasoni D., Cani D.S., Cerini M., Farina D., Gavazzi E. (2020). Cardiac Involvement in, a Patient with Coronavirus Disease 2019 (COVID-19). JAMA Cardiol..

[8] Filgueira T.O., Castoldi A., Santos L.E.R., de Amorim G.J., de Sousa Fernandes M.S., Anastacio W.L.D.N., Campos E.Z., Santos T.M., Souto F.O. (2021). The Relevance of a Physical Active Lifestyle and Physical Fitness on Immune Defense: Mitigating Disease Burden, With Focus on COVID-19 Consequences. Front. Immunol..

[9] Bowden Davies K.A., Pickles S., Sprung V.S., Kemp G.J., Alam U., Moore D.R., Tahrani A.A., Cuthbertson D.J. (2019). Reduced physical activity in young and older adults: Metabolic and musculoskeletal implications. Ther. Adv. Endocrinol. Metab..

[10] World Health Organization Support for Rehabilitation Self-Management after COVID-19—Related Illness. https://apps.who.int/iris/bitstream/handle/10665/333287/WHO-EURO-2020-855-40590-54571-eng.pdf?sequence=1&isAllowed=y.

[11] Gawrych M. (2020). Mental health during COVID-19 pandemic—A literature review. Psychiatry Clin. Psychol..

[12] Vindegaard N., Benros M.E. (2020). COVID-19 pandemic and mental health consequences: Systematic review of the current evidence. Brain. Behav. Immun..

[13] World Health Organization: Global Health Risks Mortality and Burden of Disease Attributable to Selected Major Risks. https://apps.who.int/iris/bitstream/handle/10665/44203/9789241563871_eng.pdf?sequence=1&isAllowed=y.

[14] Worls Health Organization Global Recommendations on Physical Activity for Health. https://apps.who.int/iris/bitstream/handle/10665/44399/9789241599979_eng.pdf;jssionid=FD7114E28AC829CB38889034AF5A6E92?sequence=1.

[15] Laddu D.R., Lavie C.J., Phillips S.A., Arena R. (2021). Physical activity for immunity protection: Inoculating populations with healthy living medicine in preparation for the next pandemic. Prog. Cardiovasc. Dis..

[16] Hermassi S., Bouhafs E.G., Bragazzi N.L., Ichimura S., Alsharji K.E., Hayes L.D., Schwesig R. (2021). Effects of Home Confinement on the Intensity of Physical Activity during the COVID-19 Outbreak in Team Handball According to Country, Gender, Competition Level, and Playing Position: A Worldwide Study. Int. J. Environ. Res. Public Health.

[17] Mann R.H., Clift B.C., Boykoff J., Bekker S. (2020). Athletes as community; athletes in community: Covid-19, sporting mega-events and athlete health protection. Br. J. Sports Med..

[18] Donnelly P. (2020). We are the games: The COVID-19 pandemic and athletes’ voices. Sociol. Deporte.

[19] Page M.J., McKenzie J.E., Bossuyt P.M., Boutron I., Hoffmann T.C., Mulrow C.D., Shamseer L., Tetzlaff J.M., Akl E.A., Brennan S.E. (2021). The PRISMA 2020 statement: An updated guideline for reporting systematic reviews. BMJ.

[20] WHO Definitions: Emergencies. https://www.who.int/hac/about/definitions/en/.

[21] WHO What Is a Pandemic?. https://www.who.int/csr/disease/swineflu/frequently_asked_questions/%20pandemic/en/.

[22] Moola S., Munn Z., Tufanaru C., Aromataris E., Sears K., Sfetcu R., Currie M., Lisy K., Qureshi R., Mattis P. (2020). Systematic Reviews of Etiology and Risk. JBI Manual for Evidence Synthesis. Critical Appraisal Checklist for Analytical Cross Sectional Studies. https://wiki.jbi.global/display/MANUAL/Appendix+7.5+Critical+appraisal+checklist+for+analytical+cross-sectional+studies.

[23] Moola S., Munn Z., Tufanaru C., Aromataris E., Sears K., Sfetcu R., Currie M., Lisy K., Qureshi R., Mattis P. (2020). Systematic Reviews of Etiology and Risk. JBI Manual for Evidence Synthesis. Critical Appraisal Checklist for Cohort Studies. https://wiki.jbi.global/display/MANUAL/Appendix+7.1++Critical+appraisal+checklist+for+cohort+studies.

[24] Donmez G., Ozkan O., Menderes Y., Torgutalp S.S., Karacoban L., Denerel N., Kudas S. (2021). The effects of home confinement on physical activity level and mental status in professional football players during COVID-19 outbreak. Physician Sportsmed..

[25] Villaseca-Vicuna R., Perez-Contreras J., Merino-Munoz P., Gonzalez-Jurado J., Aedo-Munoz E. (2021). Effects of COVID-19 confinement measures on training loads and the level of well-being in players from Chile women’s national soccer team. Rev. Fac. Med..

[26] Urbański P., Szeliga Ł., Tasiemski T. (2021). Impact of COVID-19 pandemic on athletes with disabilities preparing for the Paralympic Games in Tokyo. BMC Res. Notes.

[27] Mon-Lopez D., García-Aliaga A., Bartolome A.G., Solana D.M. (2020). How has COVID-19 modified training and mood in professional and non-professional football players?. Physiol. Behav..

[28] Facer-Childs E.R., Hoffman D., Tran J.N., Drummond S.P.A., Rajaratnam S.M.W. (2021). Sleep and mental health in athletes during COVID-19 lockdown. Sleep.

[29] Pillay L., Janse van Rensburg D.C., Jansen van Rensburg A., Ramagole D.A., Holtzhausen L., Dijkstra H.P., Cronje T. (2020). Nowhere to hide: The significant impact of coronavirus disease 2019 (COVID-19) measures on elite and semi-elite South African athletes. J. Sci. Med. Sport.

[30] Mehrsafar A.H., Zadeh A.M., Gazerani P., Jaenes Sanchez J.C., Nejat M., Tabesh M.R., Abolhasani M. (2021). Mental Health Status, Life Satisfaction, and Mood State of Elite Athletes During the COVID-19 Pandemic: A Follow-Up Study in the Phases of Home Confinement, Reopening, and Semi-Lockdown Condition. Front. Psychol..

[31] Parm U., Aluoja A., Tomingas T., Tamm A.L. (2021). Impact of the COVID-19 Pandemic on Estonian Elite Athletes: Survey on Mental Health Characteristics, Training Conditions, Competition Possibilities, and Perception of Supportiveness. Int. J. Environ. Res. Public Health.

[32] Ambroży T., Rydzik Ł., Obmiński Z., Klimek A.T., Serafin N., Litwiniuk A. (2021). The Impact of Reduced Training Activity of Elite Kickboxers on Physical Fitness, Body Build, and Performance during Competitions. Int. J. Environ. Res. Public Health.

[33] Roberts R.J., Lane A.M. (2021). Mood Responses and Regulation Strategies Used During COVID-19 Among Boxers and Coaches. Front. Psychol..

[34] Jaenes Sanchez J.C., Alarcon Rubio D., Trujillo M., Penaloza Gomez R., Mehrsafar A.H., Chirico A., Giancamilli F., Lucidi F. (2021). Emotional Reactions and Adaptation to COVID-19 Lockdown (or Confinement) by Spanish Competitive Athletes: Some Lesson for the Future. Front. Psychol..

[35] Di Cagno A., Buonsenso A., Baralla F., Grazioli E., Di Martino G., Lecce E., Calcagno G., Fiorilli G. (2020). Psychological Impact of the Quarantine-Induced Stress during the Coronavirus (COVID-19) Outbreak among Italian Athletes. Int J Environ Res Public Health.

[36] Fiorilli G., Grazioli E.A., Buonsenso A., Di Martino G., Despina T., Calcagno G., di Cagno A. (2021). A national COVID-19 quarantine survey and its impact on the Italian sports community: Implications and recommendations. PLoS ONE.

[37] McLean B.D., Coutts A.J., Kelly V., McGuigan M.R., Cormack S.J. (2010). Neuromuscular, endocrine, and perceptual fatigue responses during different length between-match microcycles in professional rugby league players. Int. J. Sports. Physiol. Perform..

[38] Hakansson A., Moesch K., Jonsson C., Kentta G. (2021). Affiliations expand Potentially Prolonged Psychological Distress from Postponed Olympic and Paralympic Games during COVID-19—Career Uncertainty in Elite Athletes. Int. J. Environ. Res. Public Health..

[39] Tayech A., Mejri M.A., Makhlouf I., Mathlouthi A., Behm D.G., Chaouachi A. (2020). Second Wave of COVID-19 Global Pandemic and Athletes’ Confinement: Recommendations to Better Manage and Optimize the Modified Lifestyle. Int. J. Environ. Res. Public Health..

[40] Di Fronso S., Costa S., Montesano C., Di Gruttola F., Ciofi E.G., Morgilli L., Robazza C., Bertollo M. (2020). The effects of COVID-19 pandemic on perceived stress and psychobiosocial states in Italian athletes. Int. J. Sport Exerc. Psychol..

[41] Hakansson A., Jonsson C., Kentta G. (2020). Psychological Distress and Problem Gambling in Elite Athletes during COVID-19 Restrictions—A Web Survey in Top Leagues of Three Sports during the Pandemic. Int. J. Environ. Res. Public Health.

[42] Jukic I., Calleja-Gonzalez J., Cos F., Cuzzolin F., Olmo J., Terrados N., Njaradi N., Sassi R., Requena B., Milanovic L. (2020). Strategies and Solutions for Team Sports Athletes in Isolation due to COVID-19. Sports.

[43] Sarto F., Impellizzeri F.M., Sporri J., Porcelli S., Olmo J., Requena B., Suarez-Arrones L., Arundale A., Bilsborough J., Buchheit M. (2020). Impact of potential physiological changes due to COVID-19 home confinement on athlete health protection in elite sports: A call for awareness in sports programming. Sports Med..

[44] Mon-Lopez D., de la Rubia Riaza A., Galan M.H., Refojo Roman I. (2020). The Impact of Covid-19 and the Effect of Psychological Factors on Training Conditions of Handball Players. Int. J. Environ. Res. Public Health.

[45] Eirale C., Bisciotti G., Corsini A., Baudot C., Saillant G., Chalabi H. (2020). Medical recommendations for home-confined footballers’ training during the COVID-19 pandemic: From evidence to practical application. Biol. Sport..

[46] Paoli A., Musumeci G. (2020). Elite Athletes and COVID-19 Lockdown: Future Health Concerns for an Entire Sector. J. Funct. Morphol. Kinesiol..

[47] Stokes K.A., Jones B., Bennett M., Close G.L., Gill N., Hull J.H., Kasper A.M., Kemp S.P.T., Mellalieu S.D., Peirce N. (2020). Returning to Play after Prolonged Training Restrictions in Professional Collision Sports. Int. J. Sports Med..

[48] Reardon C.L., Bindra A., Bindra A., Blauwet C., Budgett R., Campriani N., Currie A., Gouttebarge V., McDuff D., Mountjoy M. (2021). Mental health management of elite athletes during COVID-19: A narrative review and recommendations. Br. J. Sports Med..

